# Role of the central nervous system in cell non-autonomous signaling mechanisms of aging and longevity in mammals

**DOI:** 10.1186/s12576-024-00934-3

**Published:** 2024-08-31

**Authors:** Takuya Urushihata, Akiko Satoh

**Affiliations:** 1 Department of Integrative Physiology, Institute of Development, Aging and Cancer, Tohoku University, Sendai, Japan; 2 Department of Integrative Physiology, National Center for Geriatrics and Gerontology, Obu, Japan

**Keywords:** Cell non-autonomous, Aging, Longevity, Hypothalamus, Sleep, Cross-species comparison

## Abstract

Multiple organs orchestrate the maintenance of proper physiological function in organisms throughout their lifetimes. Recent studies have uncovered that aging and longevity are regulated by cell non-autonomous signaling mechanisms in several organisms. In the brain, particularly in the hypothalamus, aging and longevity are regulated by such cell non-autonomous signaling mechanisms. Several hypothalamic neurons have been identified as regulators of mammalian longevity, and manipulating them promotes lifespan extension or shortens the lifespan in rodent models. The hypothalamic structure and function are evolutionally highly conserved across species. Thus, elucidation of hypothalamic function during the aging process will shed some light on the mechanisms of aging and longevity and, thereby benefiting to human health.

## Background

Many studies have shown that longevity is regulated through cell non-autonomous signaling mechanisms by pathways originating in central nervous system neurons [1, 2]. These signaling pathways, which affect peripheral tissues, can significantly influence organismal health and longevity. Enhancement or suppression of these signaling pathways in central nervous system neurons leads to functional changes within the neurons (cell autonomous process) and transmits signals to the periphery to modulate its functions (cell non-autonomous process). For instance, in the nematode worm *Caenorhabditis elegans*, ASI amphid chemosensory neurons are important to maintain proper metabolic status, and possibly longevity. Ablation of ASI neurons completely suppresses the effect of lifespan extension induced by dietary restriction, suggesting that ASI neurons are required for the longevity effect of dietary restriction [3] through highly conserved molecules such as SKN-1 (Nrf2 homolog) and DAF-7 (transforming growth factor β homolog) [4]. SKN-1B is specifically expressed in ASI neurons. Neuronal SKN-1B, whose expression increases under dietary restriction, is required for one dietary restriction condition to promote lifespan extension [3, 5]. DAF-7 is also primarily expressed in ASI neurons. Genetic ablation of the ASI neurons prevents odorant-induced UPR^ER^ activation, depending on DAF-7 signaling, and leads to extended lifespan and enhanced clearance of toxic proteins [6]. Other signaling pathways in neurons are also reported in the regulation of nematode longevity including hypoxia-inducible factor-1 [7, 8], heat shock response proteins-1 [9], AMP-activated protein kinase (AMPK) and target of rapamycin (TOR) [10–12]. In the fly *Drosophila melanogaster*, neuronal activation of AMPK or Atg1, an autophagy-specific protein kinase, induces autophagy in the brain to slow aging and improves various parameters of healthspan [13]. Drosophila insulin-like peptides are implicated in mediating the inter-tissue responses between the nervous system and the intestines [13]. Furthermore, modifying mitochondrial function in neurons that influence aging and fly longevity also affects cells through cell non-autonomous mechanisms [14]. A recent study demonstrated that the overexpression of hedgehog signaling, which is present in the glial cells of an adult fly, rescues proteostasis defects and the reduced lifespan in the glia of hedgehog mutant flies [15]. In mammals, increasing evidence highlights the role of the brain in the regulation of aging and longevity through cell non-autonomous signaling mechanisms. Specifically, the hypothalamus stands out as  one of the most active regions involved in these signaling processes related to aging and longevity. [2, 16]. In this review, we summarize the multiple signaling pathways in the hypothalamus that convey signals from the brain to peripheral organs and modulate aging and mammalian longevity. We describe how the structure and function of the hypothalamus are conserved across species and how these aspects are altered with age. Finally, we discuss some future perspectives on aging research that focus on the hypothalamus.

## The hypothalamus in rodents

**Fig. 1:**
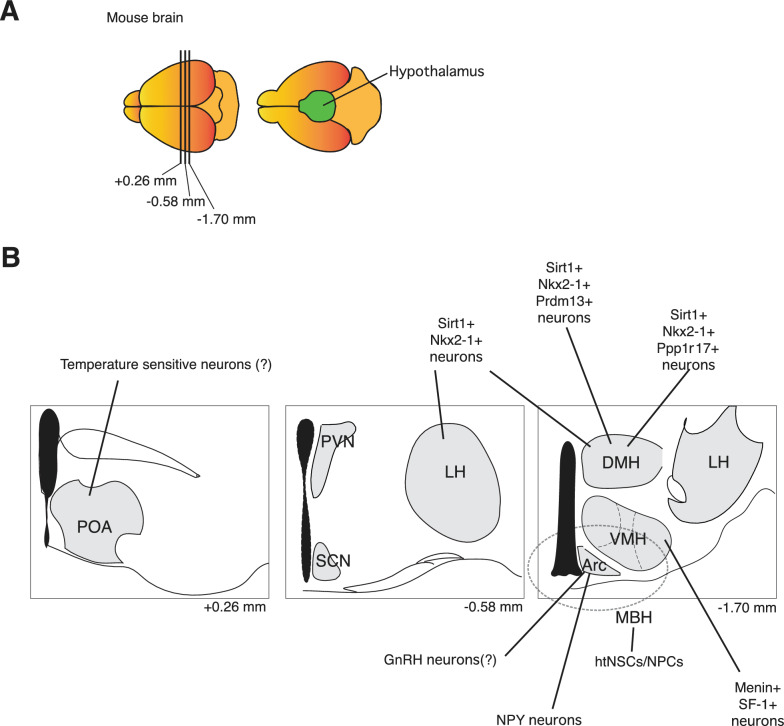
Hypothalamic neurons promote aging and longevity in rodent models. **A** Anatomical localization of the hypothalamus in the mouse brain. Three reference points are shown at Bregma + 0.26, − 0.58, and − 1.70 mm in **B**. **B** Multiple hypothalamic neurons have been reported to be involved in the regulation of aging and longevity, including temperature sensitive neurons in the preoptic area of the hypothalamus (POA); Sirt1 + Nkx2-1 +, Sirt1 + Nkx2-1 + Prdm13 +, and Sirt1 + Nkx2-1 + Ppp1r17 + neurons in the dorsomedial hypothalamus (DMH); GnRH and Npy neurons in the arcuate nucleus (Arc); hypothalamic neural stem cells/ neural progenitor cells (htNSCs/NPSs) in the mediobasal hypothalamus (MBH); and Menin + SF-1 + neurons in the ventromedial hypothalamus (VMH). PVN: paraventricular nucleus; LH: lateral hypothalamus; SCN: suprachiasmatic nucleus

In mammals, multiple longevity studies have been demonstrated lifespan extension or shortening with brain-specific manipulation of genes/signaling pathways [2, 16] (Fig. 1). The hypothalamus maintains a homeostatic balance between physiological functions and behaviors by integrating large amounts of humoral and neural information and communicating proper instructions to downstream brain regions.

An important role of the hypothalamus is humoral secretion that is potentially linked to longevity control. Ames dwarf mice and Snell dwarf mice, which are deficient in growth hormone (GH), prolactin, and thyroid stimulating hormone, live up to 40–60% longer than control mice [17, 18]. Brain-specific *insulin-like growth factor-1 (Igf-1) receptor*-knockout mice and brain-specific *insulin receptor substrate 2*-knockout mice also extend their lifespan [19, 20], indicating that inhibition of GH and insulin/IGF-1 signaling pathways within the brain increases life expectancy. Additionally, *GH-releasing hormone (Ghrh) receptor*-mutant mice live longer than controls [21]. The GHRH is mainly secreted from neuronal populations within the arcuate nucleus (Arc) and then stimulates GH secretion from the anterior pituitary. Therefore, GHRH-secreting neurons in the Arc might have a critical role in promoting the effects of GH/IGF-1 signaling pathway in longevity.

Suppression of age-associated cellular changes in the hypothalamus affects health and longevity. With age, IKKβ/NF-κB is activated in the hypothalamus [22] and the level of adult neurogenesis in the hippocampus and hypothalamus significantly declines [23]. Mice with suppression of NF-κB signaling pathway specifically in the mediobasal hypothalamus (MBH) by expression of *IκB-α* show lifespan extension [22]. Activation of NF-κB signaling in the MBH decreases *gonadotropin-releasing hormone (GnRH)* transcription, and an intracerebroventricular injection of GnRH ameliorates age-associated phenomena (e.g., muscle strength, dermal thickness, hippocampal neurogenesis, and cognitive function). In addition, multiple endocrine neoplasia type 1 (Menin) is associated with p65 and inhibits NF-κB transactivation as well as neuroinflammation. The overexpression of *Menin* in the ventromedial hypothalamus (VMH) of aged mice extends lifespan, improves learning and memory, and ameliorates aging biomarkers; whereas, inhibiting Menin in the VMH of middle-aged mice induces premature aging and accelerated cognitive decline [24]. These results suggest that neuroinflammation in the hypothalamus affects systemic aging and cognitive function. MBH-specific depletion of hypothalamic neural stem cells displays age-associated physiological changes and shortened lifespan [23]. Notably, the implantation of hypothalamic neural stem cells, expressing dominant-negative IκB-α that helps the survival of hypothalamic neural stem cells, into the MBH promotes lifespan extension. The levels of exosomal microRNAs in the cerebrospinal fluid significantly decline with age, revealing that hypothalamic neural stem cells control age-associated pathophysiology regulated by hypothalamic microRNAs [23]. Cellular senescence, defined by increases in p15, p16ink4a, senescence associated-β-galactosidase staining and DNA damage, occurs in neural stem cells/neural progenitor cells both in vitro and in vivo [2]. Such aged neural stem cells/ neural progenitor cells can be rejuvenated to induce functional neurogenesis [25], proposing a way to treat age-associated neurological diseases.

Evidence suggests that manipulating longevity-regulating genes specific to the hypothalamus, such as sirtuin and mammalian TOR (mTOR) signaling, impacts longevity. This highlights the critical role of the hypothalamus in regulating aging and lifespan. Brain-specific *Sirt1*-overexpressing transgenic (BRASTO) mice show lifespan extension in both males and females [26]. Remarkably, the aging phenomenon is ameliorated by the overexpression of *Sirt1* in the dorsomedial hypothalamus (DMH) [26]. Thus, maintaining Sirt1 signaling in the DMH might be crucial to delay the aging process and to extend our health and longevity. Supporting this idea, DMH-specific *PR-domain containing protein 13 (Prdm13)*-knockdown mice shorten the lifespan [27]. Prdm13 is a downstream gene of Sirt1 in the hypothalamus [28]. Intriguingly, DR suppresses age-associated sleep fragmentation in wild-type mice, but not DMH-specific *Prdm13*-knockdown mice, revealing that a deficiency of *Prdm13* in the DMH is sufficient to lose the effect of diet restriction in age-associated sleep alterations. Therefore, Sirt1/Prdm13 signaling in the DMH might regulate aging and longevity through sleep control. Furthermore, in the DMH, the chemogenetic activation of protein phosphatase 1 regulatory subunit 17 (Ppp1r17) ameliorates age-associated dysfunction in the white adipose tissue (WAT), increases physical activity, and extends lifespan. These findings suggest the importance of the inter-tissue communication between the hypothalamus and WAT in mammalian longevity control [29]. Although whether Sirt1 affects the function of Ppp1r17 still needs elucidation, it is conceivable that Ppp1r17+ DMH neurons can manipulate aging and longevity in mammals. Mice lacking hypothalamic mTORC2 signaling, due to the knockout of *Rictor,* show adverse effects in glucose metabolism and a shortened lifespan. In addition, hypothalamic-specific *Rictor*-knockout mice show low physical activity and increased susceptibility to diet-induced obesity through hyperphagia [30]. Only chronic administration of rapamycin inhibits mTORC2 in some cell lines or tissues [31]; therefore, specific inhibition of mTORC1 might significantly reduce the side effects of rapamycin in brain function.

Longevity studies in mice indicate a potential link between sleep control, thermoregulation, and longevity. The hypothalamus has a central role in the regulation of sleep. Sleep deficiency can be linked to many health problems including obesity, diabetes, cardiovascular disease, cognitive impairments, mental deficits, and potentially affects lifespan. In fact, young DMH-specific *Prdm13*-knockdown mice exhibited sleep alterations (e.g., sleep fragmentation, excessive sleepiness during sleep deprivation), which are similar with aged mice, and shorten their lifespan [27]. Given that restoration of Prdm13 in the DMH ameliorates age-associated sleep fragmentation, it would be intriguing to investigate whether this restoration also has an impact on longevity. The hypothalamus also acts as a control center of thermoregulation. Brain-specific *uncoupling protein 2 (Ucp2)-*overexpressing transgenic mice exhibited a lowered core body temperature by elevating the temperature within the hypothalamus, and extends lifespan [32]. Recent study indicates that, under laboratory conditions, lifespan was influenced by the level of body temperature, but not metabolic rate in both sexes in mice and hamsters [33]. In addition to low body temperature, proper adaptation of body temperature to environmental stimuli might be important to improve our health and longevity [34]. If this hypothesis holds true, *Ucp2* transgenic mice might greatly response and adjust their body temperature under low-nutrient conditions or other circumstances.

## Hypothalamic function in rodents and humans

Experiments using rodents are useful for studying the changes in brain function with age and diseases in humans; however, cross-species comparison is challenging due to discrepancies in anatomical definition and the obvious difference in brain size [35, 36]. Topologically, the hypothalamus is a known-multinucleated structure that is highly conserved across species [37–39], presumably due to its important role in animal physiology. In terms of hypothalamic neurons, single-cell transcriptomic data from the hypothalamus suggest extensive conservation of neuronal subtypes, despite certain differences in species-enriched gene expression between mice and humans [40] or between mice and macaques [41]. Furthermore, new methods for aligning measures of brain-wide gene expression in the mouse and human brains have improved the resolution of cross-species correspondences [42]. Together, elucidation of age-associated changes in hypothalamic function in rodent models will provide an insight into human aging and longevity.

Functional connectivity (FC) refers to the synchronization and/or correlation in the levels of brain activity between distinct regions of the brain. This connectivity is often assessed using functional MRI (fMRI) studies, which measure the spontaneous blood oxygen level-dependent signal. Based on structural MRI, specific regions of interest can be defined as a seed [43] that allows to measure the regional FC. An entire brain functional network is further estimated by fMRI. The human brain functional network alters in several physiological dysfunctions including mild cognitive impairment [44], neurodegenerative diseases (for example, Alzheimer's disease [45, 46], Parkinson’s disease [47], Huntington’s disease [48]), schizophrenia [49], depression [50] and insomnia [51], suggesting that the determination of regional FC is useful for the diagnostics of brain function. Some FCs are highly conserved across species (for example, visual and somatomotor networks), while some regions appear to be unique and unassigned [36, 52].

## Functional and structural network involving the hypothalamus and its alterations with age

As individuals age, the functional network of entire human brain, which is well-segregated and specialized in young individuals, undergoes deterioration (for example, decreased FC within functional networks and increased FC between functional networks). Such age-related changes are pronounced across multiple brain networks, with particular attention given to the default mode network in previous studies [53–57]. In rodent studies, an age-related decline in FC within networks is consistent with that in human studies; however, age-related changes in connectivity between networks are not fully elucidated. So far, a few studies have demonstrated age-related changes in seed-based FC of the hypothalamus in mice and humans. In mice, the FC of the hypothalamus with the prelimbic cingulate and hippocampus significantly increases from 2.5 to 8.5 months of age. In contrast, the connectivity with the globus pallidus and prelimbic cingulate decreases from 8.5 to 12.5 months of age, creating a distinct  inverse U-shaped curve) [58]. Further, evaluation of FC of the hypothalamus in rodents aged at 12.5 months and older would be valuable. Notably, a similar inverse U-shaped curve is also reported in humans [59]. In humans, no study has shown distinct age-related changes in seed-based FC of the hypothalamus [60]. However, a reduction in FC between the hypothalamus and temporal gyrus is observed in patients with Alzheimer's disease accompanied by depression [61]. Intriguingly, female rats have a stronger hypothalamic-related connectivity than males, suggesting a sexual dimorphism of hypothalamic FC [27].

Structural connections have been investigated for the hypothalamus and its changes with diseases and aging using a diffusion MRI-based technique, called diffusion tensor tractography, in humans and using neurotracing studies in rodents. The diffusion tensor tractography can non-invasively depict the trajectory of a neural fiber tract [62], and provides information about anatomical connections between distant brain areas and the course, interruption, or integrity of neural pathways [63]. Aging causes a decrease in the structural connectivity density and the total number of fiber tracts in the brain [56]. Moreover, patients with mild traumatic brain injury (MTBI) show disrupted structural connections within the hypothalamus, and reduced FC between the hypothalamus and the medial prefrontal, inferior posterior parietal, or cingulate regions [64]. MTBI can cause injury to hypothalamic cell bodies that may result in substantial neuropeptide dysregulation with associated clinical symptoms, including motivated behavior, sleep/wake cycles, and arousal. Thus, addressing whether sleep disturbance significantly affects FC changes within the hypothalamus, and whether it is impacted by age would be valuable. Mouse structural connectivity in the hypothalamus revealed by neurotracing studies can be found in the Allen Mouse Brain Connectivity Atlas [65]. Projections from the lateral hypothalamus to the zona incerta, reticular nucleus of the thalamus and perifornical nucleus are commonly reported in multiple studies. Rodent studies have reported fiber injury with age in the thalamus and cortex, but not in the hypothalamus [66].

## Conclusion

In mammals, certain neurons in the hypothalamic nuclei play a crucial role in regulating physiology against aging and prolong lifespan. To further elucidate hypothalamic mechanisms of aging and longevity via cell non-autonomous pathway, future studies can address the following issues: (1) whether the identified hypothalamic neurons interact with each other in regulating of mammalian longevity; (2) whether there is a key connection between the hypothalamus and the external/internal regions of the brain; and (3) whether the FC between hypothalamic nuclei changes with age. This is challenging to address due to the resolution limit of MRI; however, other techniques might be feasible to address this question.

## Data Availability

Not applicable.
